# Tumor infiltrating lymphocytes as an endpoint in cancer vaccine trials

**DOI:** 10.3389/fimmu.2023.1090533

**Published:** 2023-03-07

**Authors:** Patrick M. McCarthy, Franklin A. Valdera, Todd R. Smolinsky, Alexandra M. Adams, Anne E. O’Shea, Katryna K. Thomas, Spencer Van Decar, Elizabeth L. Carpenter, Ankur Tiwari, John W. Myers, Diane F. Hale, Timothy J. Vreeland, George E. Peoples, Alex Stojadinovic, Guy T. Clifton

**Affiliations:** ^1^ Department of Surgery, Brooke Army Medical Center, Ft. Sam Houston, TX, United States; ^2^ Department of Surgery, University of Texas Health Science Center, San Antonio, TX, United States; ^3^ Department of Surgery, Madigan Army Medical Center, Ft. Lewis, WA, United States; ^4^ Cancer Insight, San Antonio, TX, United States

**Keywords:** tumor infiltrating lymphocyte (TIL), cancer vaccine, vaccination, endpoint, immunotherapy, checkpoint inhibition

## Abstract

Checkpoint inhibitors have invigorated cancer immunotherapy research, including cancer vaccination. Classic early phase trial design and endpoints used in developing chemotherapy are not suited for evaluating all forms of cancer treatment. Peripheral T cell response dynamics have demonstrated inconsistency in assessing the efficacy of cancer vaccination. Tumor infiltrating lymphocytes (TILs), reflect the local tumor microenvironment and may prove a superior endpoint in cancer vaccination trials. Cancer vaccines may also promote success in combination immunotherapy treatment of weakly immunogenic tumors. This review explores the impact of TILs as an endpoint for cancer vaccination in multiple malignancies, summarizes the current literature regarding TILs analysis, and discusses the challenges of providing validity and a standardized implementation of this approach.

## Novel immunotherapy biomarkers needed

Historically, novel chemotherapy agents have first been tested in patients with advanced disease and few efficacious treatment options. These early phase trials help determine safe dosing regimens and toxicity profiles but may also give a signal of therapeutic efficacy. Drugs with acceptable toxicities and evidence of effectiveness then advance to later phase testing in patients with a lower disease burden. Because effective cytotoxic chemotherapies have historically shown objective responses in at least a subset of advanced or metastatic cancer patients, early phase trials in patients with advanced disease will generally give a signal of potential therapeutic efficacy relatively quickly and with few patients (1, 2). This strategy may not be as effective, however, for all therapies.

The revolutionary success of immune checkpoint inhibitors (ICIs) in the treatment of certain malignancies has invigorated cancer immunotherapy research. As this research has progressed, it has become clear that classical early phase trial design and endpoints used for cytotoxic chemotherapy are not always appropriate for immunotherapy trials (3, 4). Cancer vaccines, particularly as monotherapy, seem to be more effective in the setting of low disease burden, and may not produce the dramatic radiographic responses seen with cytotoxic therapy (5). Thus, cancer vaccine development may struggle to show effectiveness when studied in late-stage disease, and when traditional assessment methods are used (3, 6). Without the use of these traditional endpoints, such as treatment response determined by standard radiographic response evaluation criteria, novel biomarkers are needed to meaningfully determine which cancer vaccines should progress out of early phase trials and into larger phase III/registrational clinical trials.

Failures of traditional endpoints in cancer vaccine clinical trials have led investigators to rely on immunologic surrogates to signal efficacy in early-phase vaccine trials (3). The immune response to cancer vaccines has traditionally been measured with T-cell assays, presuming that measuring T cell responses in peripheral blood will predict the immune system’s response to the tumor locally. This has been accomplished with techniques such as HLA-peptide multimer staining assays, enzyme-linked immunosorbent spot (ELISPOT), intracellular cytokine staining, and peripheral blood cytokine levels to quantify immune response (7–9). Unfortunately, these assays have significant variability and have historically proven unreliable in predicting clinical efficacy; in part because the T cell response in the peripheral blood does not accurately reflect the activity of T cells within the tumor microenvironment (TME) (10, 11). The absence of exploratory biomarker guidance by the Food and Drug Administration (FDA) is telling. In their 2011 guidance on therapeutic cancer vaccines, the FDA states that recommendations for the most appropriate early endpoints are immature, and more recent guidelines have not been offered (12).

As an alternative to peripheral blood assays, recent evidence suggests that measurable immunologic changes within the TME are more reflective of how the vaccine affects the tumor and may serve as a better surrogate for clinical effectiveness (9, 13–16). Specifically, the measurement of T cell infiltration within the TME may be the most direct way to assess the immunologic effect of vaccination and may help predict synergistic effect when in combination with other therapies such as ICI. In this review, we explore the experiences of assessing tumor-infiltrating lymphocytes (TILs) in response to vaccination across multiple malignancies, summarize the current body of literature on the understanding of TILs evaluation as a marker of prognosis, and discuss the remaining challenges facing validation and widespread adoption of this approach.

## Tumor infiltrating lymphocytes

**Figure 1:**
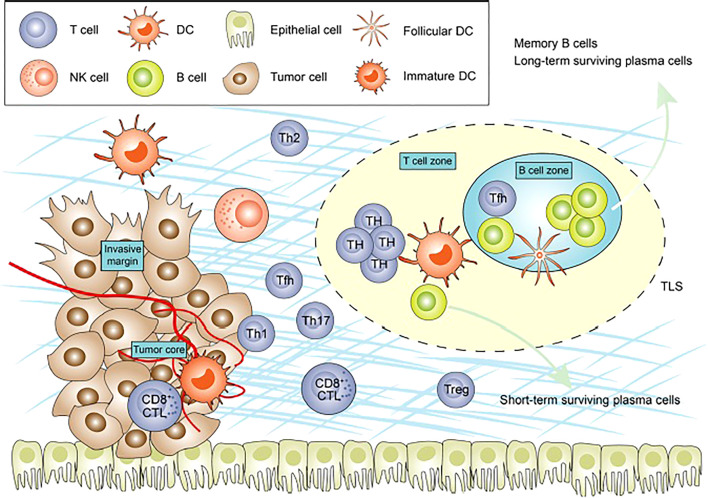
Figure adapted from recent Frontiers publication by Bai et al. demonstrating the cellular composition of the tumor microenvironment.

TILs are lymphocytes that have left the bloodstream, migrated to a tumor, and are at times able to recognize and kill cancerous cells (17). At the site of the tumor, subsets of TILs interact with each other and with other immunologically active cells in complex, symbiotic, and antagonistic ways to form the TME (**Figure 1**) (18). The location, quantity, function, and ratio of TIL subsets helps to characterize the hosts’ immune response to cancer. In general, increased numbers of TILs is associated with superior clinical outcomes, but a more sophisticated understanding of location and subpopulations may improve our understanding of both the tumor-host immune interaction, and our ability to determine prognosis and guide treatment (19, 20).

One such way to standardize assessments of TILs is through objective scoring systems, such as Immunoscore. Immunoscore is a novel staging method incorporating the type, density, and location of immune cells within tumor samples, which has been validated in colon cancer (9). Immunoscore is based on the numeration of cytotoxic CD3+ (CD45RO+ in a prior iteration) and CD8+ lymphocytes along with their locations in the tumor center or at the invasive margin. Scoring is from 0 to 4, with Immunoscore 0 (I0) corresponding to low densities of both cell types, and Immunoscore 4 (I4) corresponding to high densities of each (21). The prognostic importance of this scoring method was validated in patients with stage I and II colorectal cancer, where significant improvements in disease-free, disease-specific, and overall survival was correlated with increasing quantities of CD8+ and CD45RO+ immune cells in the tumor center and invasive margin (15). Similar results were obtained in a trial of patients with stage II and III colon cancer (22). Recognizing TILs as a surrogate of tumor antigenicity, pathologists have proposed standardized, routine reporting methods (23, 24). While standardized TIL analyses are being increasingly used in both prognostic and predictive roles for multiple solid malignancies, the optimal assessment of the TME remains uncertain (25–27).

The importance of TILs is also underscored by the ability to predict treatment response to ICI therapy based on their density and location (28). ICIs are now delivering durable responses in diseases where traditional chemotherapy had previously failed, such as melanoma, non-small cell lung cancer (NSCLC), renal cell carcinoma (RCC), urothelial cancer, cancers with high-level microsatellite instability (MSI-H), or triple negative breast cancer (TNBC) (29). Tumors with greater number of TILs within the TME, often termed “inflamed” tumors, are more likely to respond to ICI therapy (30).

The prognostic value of TILs in untreated tumors and after chemotherapy is well established. Additionally, the value of a change in TILs within the TME as a response to cancer immunotherapy has also been demonstrated with immune checkpoint inhibition where an increase in TILs after treatment is prognostic, correlating with an improved survival outcome (31). The prognostic value of assessing TILs in the TME in patients receiving therapeutic vaccines is another interesting possible application that would need to be further studied in future studies.

ICIs may not be as effective, however, in non-immunogenic or weakly immunogenic tumors. In such situations, cancer vaccines may elicit an antigen-specific T-lymphocyte response that leads to infiltration of TILs into the TME (32). Vaccination has also been shown to increase the expression of PD-1, marking an expanded immune response in tumors (33). With increased immunogenicity through vaccination, ICIs may become successful in weakly immunogenic tumors, where they had previously failed. While there is a theoretical concern that vaccination with ICI could lead to increased immune-related adverse events (AEs), this has not been observed in published trials to date (34). However, this is something that will continue to be necessary in trials combining vaccines with ICIs, Immunoscore has been identified as prognostic in untreated colon cancer, but an analogous scoring system for increased infiltration of TILs after vaccination would be useful for understanding the potential efficacy of vaccine therapy.

## Methods for functional characterization of lymphocytes within TME

To understand the TME, and changes therein, it is necessary to identify and quantify immune cell subtypes within the TME. Evaluation of the immune TME continues to rapidly evolve as the technology improves and our understanding of interactions between cancer and the immune system advance. Below is a brief discussion of some of the currently used methods to evaluate the immune TME.

Immunohistochemistry (IHC) is a well-established technique to analyze the qualitative and quantitative aspects of protein expression and biomarker identification. This evaluation serves as the basis for diagnosis and has been utilized to identify cell types within tissues, such as the variety and quantity of TILs within the TME. Flow cytometry is used to analyze cellular marker expression and subsequently characterize cell subtypes within tumor and surrounding tissue samples. These approaches have long been utilized for the purposes of diagnosis. However, they present a challenge when seeking to identify more than one marker per tissue section. This has improved with the advent of multiplex IHC and immunofluorescence (IF) where multiple markers can be identified in addition to detailing the spatial relationship of cells or labeled markers relative to other structures within tissue—a distinction that has become valuable in cancer immunotherapy and defining the tumor and surrounding cellular landscape (35). With spatial relationships, cell location within the TME is identified and can help stratify which cell lines are in the tumor center, tumor invasive margin, or on the periphery.

The innovation of single-cell RNA sequencing allows the categorization of individual TIL subsets as well as novel markers, improving the ability to discriminate particular immune subsets within the TME by RNA expression (36). Multiple gene expression and quantification tools used for bulk RNA sequencing have been adapted for single-cell RNA sequencing approaches, including Spliced Transcripts Alignment to a Reference (STAR), RNA-seq by Expectation-Maximization (RSEM), and the RNA-seq quantification program Kallisto. The Estimation of Stromal and Immune cells in Malignant Tumor tissues using Expression data (ESTIMATE) algorithm can be used with single-cell RNA sequencing to identify tumor phenotype and even the proportion of tumor, immune, or stromal cells (37–39). Other novel methods of cell subtype identification, include Cell-type Identification By Estimating Relative Subsets Of RNA Transcripts (CIBERSORT), a method for characterizing cell composition of complex tissues from their gene expression profiles using a database reference to identify RNA, DNA, and other cellular component mixtures from a variety of tissues. This approach demonstrates improved accuracy in cases of unknown tissue content or closely related cell types (40, 41). Though these methods allow for a more in-depth look into the cell transcriptome, they are unable to answer questions relating to the larger TME structure or cell surface expression (42). Methods such as spatial transcriptomics however seek to resolve this drawback, presenting promising outlooks on characterizing tumor heterogeneity in the context of spatial properties and how these relationships correlate with cancer treatment and survival (43).

Immunoseq is a similar tool designed to allow efficient re-sequencing of regulatory regions of relevance in immune cells (coding and non-coding) and thus enable a comprehensive assessment of all potentially relevant variation in these regions, both common and rare. This strategy is rooted in observations from genome-wide association studies that genetic variants are frequently found in non-coding regions of the genome; suggesting that altered gene expression determines many complex traits. With this approach, a more practical and affordable method of identifying both coding and non-coding variations can be accomplished. Specifically, non-coding regions have been found to often modify transcription factor binding patterns. These findings could aid in narrowing the variations which are involved with the development of immunologic and inflammatory diseases (44, 45).

While the analysis and quantification of TILs genetic and epigenetic data can provide detailed information about the subtype and activity levels of these cells, the multitude of available technologies highlights the large variety of methods utilized and need for an evidence-based, standardized utilization approach to reliably predict therapeutic effectiveness. Regardless of method used, evaluation of the functional status of cytotoxic and helper T cells, and quantification of immunosuppressive populations provides a deeper understanding of the status of the immune TME and its capacity to suppress or eliminate cancer cells and response to IO-based therapies. Additionally, evaluating for targetable immune checkpoints, such as PD-1,PD-L1 and CTLA-4 may help identify opportunities for combinations with novel or existing targeted therapies that could show synergistic results to overcome treatment resistance and improve patient outcomes. As TIL assessment and reporting becomes more prevalent in oncologic research and clinical use, a more standardized approach to evaluate the TME will aid in directly comparing results between studies.

## TILs measured in cancer vaccine trials

### Metastatic melanoma

A phase 1 trial was performed in 18 patients with advanced, unresectable stage IIIC or IV melanoma who received prime boost intra-nodal injections of MKC1106-T (the plasmid pMEL-TYR and two peptides corresponding to melan-A and tyrosinase) (46). Peripheral immune response was measured by MHC tetramer analysis, ELISPOT assays, and evaluation of persistent pMEL-TYR plasmid levels in the blood by PCR. Expression of target antigens melan-A, tyrosinase, and beta-2 microglobulin were determined in pre-immunization tumor biopsy specimens by IHC. A significant peripheral immune response was defined as greater than a 2-fold increase in the tetramer assay or 3-fold ELISPOT assay from baseline.

Of the 18 patients enrolled, only 14 patients (78%) were able to be evaluated for immunologic response as 4 patients did not complete treatment due to adverse events. Of these 14, nine patients (64% by per-treatment analysis) demonstrated a significant peripheral immune response to the target antigens melan-A or tyrosinase. Four patients remained without disease progression for a minimum of six months and demonstrated radiologic evidence of tumor regression, with only two patients showing an increase in antigen-specific T-cells against both antigens. Overall, disease control did not correlate with expression of circulating antigen-specific T-cells in the blood; however, there was a correlation found between pre-existing melan-A specific T-cells as TILs in pre-treatment biopsies and long-term disease control. Of the four patients that experienced tumor regression, two patients underwent core needle biopsy after several immunizations. IHC and flow-cytometry analysis of these biopsies demonstrated CD8+ and CD4+ subsets of TILs with CD8+ cells being diffusely present throughout tumor that stained for the target antigens. TILs were noted to be increased in the post-treatment biopsies, but no means of comparison pre/post biopsy was built into the study. In both of these patients, CD8+ TILs specific to melan-A and tyrosinase were primarily CD27+, CD28-, CD45RA- suggesting that these T cells were effector memory T cells

### Locally recurrent or progressive prostate cancer

A phase 1 trial examined 19 patients with locally recurrent prostate cancer at least 18 months after definitive radiation therapy and three consecutively rising PSA values (47). Patients received intra-prostatic administration of PSA-TRICOM (PROSTVAC→), a prostate specific antigen (PSA)-targeted poxviral vaccine (viral vector-based IO). Pre- and post-treatment prostate biopsies were obtained to evaluate for tumor infiltrating CD3+, CD4+, and CD8+ cells. After vaccination, CD4+ cells were increased from 1.7 cells/high powered field (hpf) to 12.3 cells/hpf (p=0.0005). Similarly, CD8+ cells increased from 6.5 cells/hpf to 14 cells/hpf (p=0.0007) and were found in higher concentration throughout the epithelial areas of the tumor. Though the T-cells were not tested for tumor-specificity, when considering the reduction or stabilization in PSA of 11 of 13 patients with a simultaneous increase in TILs post-biopsy, the findings suggest a tumor-specific response.

This same cohort of patients had further characterization and correlation of their peripheral immune and local immune responses (48). While a previous phase 2 study of systemic PROSTVAC demonstrated an association between overall survival and changes in the frequency of peripheral T_regs_ and CD4+ T-cells (49), analysis of immune response by multicolor flow cytometry in this trial of systemic and intra-tumoral vaccination showed no significant change in the frequency of peripheral immune cell subsets. Although, a trend of decreased serum PSA with a lower percentage of peripheral CD4+, CD25+ T_regs_ cells (p<0.025) was noted. The authors of this trial postulate that the lack of change in peripheral immune cell frequency could be a result of intra-tumoral sequestration of immune cells as a result of intra-tumoral vaccination, i.e., an increase in TILs. This is supported by the increases in CD4+ and CD8+ TILs within the tumor and tumor stroma after vaccination as well as an observed inverse correlation between CD4+ TILs and CD4+ PMBCs post-vaccination. Furthermore, a statistically significant correlation was seen between increased post vaccine CD8+ TILs and decreases in serum PSA (p=0.002, R=-0.83).

Sipuleucel-T is a vaccine generated from autologous PMBCs cultured ex-vivo with recombinant fusion protein PA2024 composed of prostate acid phosphatase (PAP) and GM-CSF that is currently the only cancer vaccine which is FDA approved. Vaccination with Sipuleucel-T was shown to convey a significant survival benefit in two phase 3 trials of men with castrate-resistant prostate cancer (50, 51). The vaccine has been shown to generate systemic immune responses to PA2024 and PAP; however, its effect within the TME is poorly understood. The vaccine was evaluated in an open label phase 2 study of 42 patients with untreated localized prostate cancer who received Sipuleucel-T immunotherapy prior to radical prostatectomy. Sipuleucel-T was administered every 2 weeks, starting 6-7 weeks prior to prostatectomy, with post-surgical randomization to either receive or not receive a booster at 12 weeks post-operatively. Peripheral immune response was measured with (^3^H) thymidine T-cell proliferation and ELISPOT assays. Infiltrating CD3+, CD8+, and CD4+FOXP3- helper, and CD4+FOXP3+ T_regs_ cells were quantified by IHC and digital image analysis and compared between pre-treatment biopsy and prostatectomies. Of the forty-one patients who were enrolled, thirty-seven patients underwent prostatectomy, and were evaluated for peripheral immune response as well as tumor immune infiltration. In all three of the tumor areas (benign glands, tumor interface, tumor center) that were examined, there was a statistically significant increased infiltration of CD3+, CD8+, CD4+FOXP3- helper, and CD4+FOXP3+ regulatory subtypes (p<0.001). Of these, CD8+ cytotoxic T cells at the tumor interface increased from a mean of 0.65x10^-3^/µm^2^ to 2.87x10^-3^/µm^2^ (p<.001). While CD4+ FOXP3+ T_regs_ at the tumor interface increased from pre-treatment levels, their recruitment to the tumor interface was to a lesser extent than CD4+ FOXP3- or CD8+ T-cells. Interestingly, neoadjuvant Sipuleucel-T induced a significant increase in PD-1+ cells at the tumor interface; the majority of which expressed Ki-67, which is indicative of a non-exhausted phenotype (52). Using Spearman correlation, there were also statistically significant associations between CD4+ T-cells at the tumor interface with both PA2024 and PAP ELISPOT (p=0.01 and p<0.01, respectively) as well as a correlation between CD3+ TILs in benign tissue and the tumor center with PAP ELISPOT (p=0.01 for each).

The increase of TILs within the TME after neoadjuvant vaccination with Sipuleucel-T is encouraging as a higher local immune response of T cells within tumors has been associated with improved outcomes in multiple types of malignancy, such as colorectal cancer (CRC) (53). In this study, neoadjuvant Sipuleucel-T significantly increased the frequency of TILs within post-prostatectomy specimens. Although this increase of TILs had a limited correlation with the peripheral immune response, it is thought that this is due to the tumor antigen-specific T-cells localizing to the site of malignancy (54). The view that tumor specific T cells migrate from the periphery to the site of malignancy is supported by the post-treatment specimens harboring significantly increased infiltration of CD3+, CD8+, CD4+FOXP3- helper, and CD4+FOXP3+ regulatory subtypes. Further lending to the view of an augmented tumor-specific immune response after neoadjuvant vaccination is that there was also not an in increase the magnitude of natural killer (NK) cells, which would suggest a non-specific immune response, in post-treatment specimens treated with neoadjuvant Sipuleucel-T. Overall, the results of an increased infiltration of multiple lines of T cells as a response to vaccination without an elevated non-specific immune response in the TME provides evidence to support that TILs are a potentially useful biomarker of response in prostate cancer.

### Pancreatic adenocarcinoma

A phase 2 trial evaluated 59 patients with resectable pancreatic ductal adenocarcinoma (PDAC) who were randomized to receive neoadjuvant GM-CSF secreting allogeneic PDAC vaccine (GVAX) alone, GVAX plus intravenous cyclophosphamide, or GVAX plus oral cyclophosphamide. All patients received the first GVAX vaccination intradermally approximately 2 weeks before pancreaticoduodenectomy followed by standard chemotherapy and radiation therapy. Post-operatively, patients received up to five additional GVAX doses. Resected pancreatic cancer specimens were analyzed and compared to 54 unvaccinated historical control specimens. Thirty-nine patients remained on the study after others were excluded for non-PDAC pathology, metastases at surgery, and immediate recurrences. These 39 patients received standard adjuvant chemoradiotherapy and up to five additional GVAX doses, each administered every 4 weeks.

Tertiary lymphoid aggregates are ectopic lymphoid structures whose formation depends on antigen presentation. They develop in areas of inflammation with a structure resembling B- and T-cell zones within lymph nodes. Their presence suggests an augmented adaptive immune response to persisting antigens due to the placement of an entire immune response unit into a specific site, the TME in this case (55). Evaluation of resected specimens that received GVAX demonstrated the creation of intratumoral tertiary lymphoid aggregates in 85% (33/39) of patients, a finding not present in any of the 54 patient specimens collected prior to vaccination (p<0.001). IHC analysis of these aggregates revealed actively proliferating cells (Ki67+), follicular dendritic cells (CD21+), mature dendritic cells (CD83+, DC-LAMP+), and monocytes (CD68+, CD163+). The presence of these lymphoid aggregates within the tumor suggests that GVAX vaccination can induce immunogenicity in a classically “cold” tumor; an exciting finding given that lymphoid aggregates are associated with an improved prognosis in immunotherapy naive patients with a variety of tumor types (55–58). In the survival analysis, only 13 patient tumors with lymphoid aggregate data also had survival data, 10 having aggregate positive tumors and the other 3 being aggregate negative. The presence of intratumoral lymphoid aggregates was associated with improved, not statistically, significant increase in overall survival >3 years in 46% of patients (6/13) with the remaining 54% (7/13) having an overall survival of <1.5 years (p=0.069). All patients with a survival benefit had aggregate positive tumors while 43% (3/7) of those with a shorter survival were aggregate negative. While the presence of intratumoral lymphoid aggregates failed to demonstrate an absolute survival benefit, it was associated with a trend to increased overall survival (59).

Additional IHC analysis revealed the presence of immunosuppressive PD-1 and PD-L1 expression in all intratumoral lymphoid aggregates, not present in control specimens. Within these aggregates, increased levels of PD-1 expression were associated with improved survival as were decreased levels of PD-L1 expression; suggesting that PD-1 may be a marker of activated T cells in the intratumoral aggregates. Additional TIL analysis was correlated with outcomes and centered on prior experience demonstrating that Tregs infiltration is associated with poor prognosis (60, 61) and that higher T effector cell:Tregs cell ratios within tumors correlate with antitumor immunity (62–65). Given this, ratios of effector T cells to Tregs in 10 vaccinated and 3 unvaccinated specimens were compared. The ratio was represented by IFN-γ producing CD8+ effector T-cells to CD4+ FOXP3+ Tregs where the authors demonstrated a higher ratio in 70% (7/10) of patients who received GVAX and a greater than one log-fold increase in 71% (5/7) of those vaccinated patients with increased ratios relative to unvaccinated patients (p= 0.10) (59). Although not statistically significant, this provides evidence that GVAX may stimulate an anti-tumor response and improve prognosis as with other tumors that have been demonstrative of these effector T cell to regulatory T cell ratios.

### Colorectal cancer

TroVax is a highly attenuated vaccinia virus (modified vaccinia Ankara, MVA) vaccine containing the gene for the tumor-associated antigen, 5T4, under control of a modified vaccinia virus promoter, mH5. Expression of 5T4 has been associated with poor prognosis in CRC (66, 67) and the magnitude of 5T4-specific response has been shown to correlate with disease-free survival (DFS) (68). The vaccine was evaluated in a phase 2 study of 20 patients with CRC liver metastases who received two doses of TroVax prior to hepatic metastatectomy, and additional doses at four and eight weeks postoperatively. In the resected tumor specimens, TILs were observed primarily in the peritumoral areas with some TILs observed within tumor islands. TIL subtype analysis was limited but showed a primarily CD4+ infiltration relative to CD8+ (mean peritumoral concentrations of 198 cells/mm^2^ and 57 cells/mm^2^, respectively) with a mean CD4+:CD8+ ratio of 3.5:1. Fifteen of twenty patients were deemed evaluable as defined by completing metastatectomy and receiving at least 4 vaccinations. These patients were then stratified into categorical variables based on those who had above or below median proliferative responses, serologic responses, and TILs. A 5T4-specific proliferative response was associated with a significant survival advantage (p=0.05) that was not shared with an MVA-specific proliferative response (p=0.754). Additionally, the magnitude of peritumoral CD3+ cells was significantly associated with longer survival (p=0.012), but there was no survival advantage associated with CD4+ or CD8+ T-cell infiltration nor the CD4+/CD8+ TILs ratio (69).

Vermorken et. Al demonstrated improved DFS and trend towards prolonged OS with vaccination in a randomized controlled phase 3 trial enrolling stage II or III CRC who received an autologous tumor cell-Bacillus-Calmette-Guerin (BCG) vaccine in the adjuvant period or no further treatment. This study, and subsequent follow-ups of it, demonstrate a correlation of adjuvant vaccination with improved outcomes in the setting of a strong pre-existing TILs response. Vaccinations began 4 weeks after resection and were repeated for 3 consecutive weeks followed by a booster vaccination containing irradiated tumor cells at 6 months. At a median follow-up of 5 years, patients with stage II colon cancer were shown to have decreased recurrence (p = 0.023), improved recurrence free survival (p=0.032), and a trend towards improved OS (70). In a follow-up to this study, the subset of patients (n=49) with stage II microsatellite stable (MSS) tumors had an improved DFS when vaccinated (71).

An additional inspection of tissue from the original cohort was used to determine the value of TILs in prognostication and prediction of clinical outcomes in an additional 15-year follow-up. The aim of this study was to explore recurrence-free interval (RFI) and disease-specific survival (DSS), where, as stated above, stage II MSS tumors had an improved DFS, when receiving adjuvant vaccination. One hundred and six samples were evaluable by digital image analysis software, 99 of which were able to be stained for CD3+ and 104 samples for CD8+. These samples were stratified into MSI-H (n = 24), and MSS (n = 82) colon cancer. Given that MSI-H colon cancer did not have enough recovered specimens to provide subset analyses of significance, the data focused on MSS colon cancer. A subsequent survival analysis demonstrated an association between a higher invasion of CD3+ TILs within tumor stroma and an increased 5-year survival (p=0.01) for patients with stage II MSS tumors. DFS at 5 years was also associated with a higher number of CD8+ cells within the tumor nest, or intraepithelial tumor invasion, for stage II patients and stage III patients combined (72).

To further explore the influence of vaccination with the magnitude of tumor stromal TIL infiltrate, a subset analysis of MSS stage II colon cancer was performed, separating patients with high *vs* low TILs prior to surgery. A high infiltrate of CD3+ T cells within the tumor stroma prior to resection was shown to predict an improved DFS and RFS when patients received adjuvant vaccination (p = 0.01) as opposed to the control group also with high pre-operative stromal CD3+ T cells. Interestingly, in patients with low infiltrates of stomal CD3+ T cells, there was no difference in outcomes, DFS and RFS, between vaccinated and control groups. This suggests that patients with a more robust *de novo* immune response to their MSS CRC are more responsive to vaccination, as demonstrated by inferior DSS and RFI in patients who received the vaccine but had a low presence of stromal TILs in specimens. Furthermore, vaccination itself likely improves outcomes given an improved DSS and RFI with vaccination in patients with high stromal TILs (72).

While there are differences across the vaccine trials highlighted above, they provide several examples of studies that have demonstrated a change in the TME using quantification of activated T cells in response to vaccines (**Table 1**). The trials differed in subtypes of TILs evaluated but the ability to detect changes in the tumor itself in response to vaccination suggests that this may be a useful surrogate for evaluating effectiveness of the vaccine and warrants further study. This could be particularly useful for helping determine optimal dose and schedule of a vaccine in early phase trials when the numbers of evaluable patients are typically smaller. It will be important to validate the usefulness of this approach in future studies by correlating changes in TIL sub-populations with clinical effectiveness results.

**Table 1:** Previously studied TILs response measure in cancer vaccine trials.

Study	Type(s) of Cancer	Method(s) for Analyzing Immune Cells	Immune Cells Analyzed	TILs Results with Vaccination	Correlation between TILs and Clinical Aspects
(46)	Metastatic Melanoma	MHC, ELISPOT, PCR	CD8 TILs specific to melan-A and tyrosinase	64% demonstrated increase in target antigens	50% of patients with tumor regression had two fold-increase in target antigens
(47)	Prostate Cancer	IHC	CD3+, CD4+, CD8+	84% increase in TILs post-biopsy	Direct correlation between TILs increase and decreased PSA levels
(51)	Prostate Cancer	thymidine T-cell proliferation, ELISPOT, IHC	CD3+, CD8+, CD4+FOXP3-, CD4+FOXP3+	Increase in all cell lines at benign glands, tumor interface, and tumor center of biopsies	Statistically significant increased infiltration of all subtypes in post-surgery specimens in 90% of patients
(58)	Pancreatic Adenocarcinoma	IHC	Ki67+, CD21+, CD83+, DC-LAMP+, CD68+, CD163+	Increased lymphoid aggregates in 57% of post-surgery specimens	Presence of intratumoral lymphoid aggregates was associated with improved, not statistically, significant increase in overall survival >3 years in 46% of patients
(68)	Metastatic Colorectal Cancer	IHC	CD4+, CD8+, 5T4 tumor-associated antigen	Increase in TILs in primarily peri-humoral areas with CD4+ predominance	5T4-specific proliferative response was associated with survival advantage
(71)	Colorectal Cancer	IHC	CD3+, CD8+	Increased invasion of CD3+ and CD8+ TILs within tumor stroma	Increased 5 year survival and DFS in stage II MSS tumors

## Challenges in assessing TILS in TME

There are logistical problems to obtaining and assessing paired or serial tumor biopsies for TILs in clinical trials, making patient selection and clinical trial design essential. The need to safely obtain multiple biopsies restricts the ideal patient population to individuals with tumors that are typically amenable to core needle biopsies, such as CRC liver metastases, breast cancer, and melanoma. Additionally, the success rate of obtaining paired, evaluable tumor biopsies from patients in clinical trials is typically 70% or less—trials need to be powered to account for this (73, 74). There is also a degree of heterogeneity within the TME, which creates the possibility that differences observed between tumor biopsies due to heterogeneity could be falsely attributed to treatment effect as opposed to the tissue sampling technique utilized (75). Obtaining paired biopsies from the same primary or metastatic same, collecting multiple cores with each biopsy, evaluating TILs within the entirety of the evaluable tumor sample, and appropriately powering studies to account for variability all help to address the challenge of tumor heterogeneity.

## Discussion

TILs have been examined in multiple vaccine trials (46–48, 52, 59, 76–78) and these investigations support the ability of vaccination to alter the frequency and composition of TILs within the TME when compared pre- to post-treatment. Generally, changes in TILs have not correlated well with changes in the circulating immunity in response to vaccine treatment (46, 48, 59). This raises the possibility that changes to TILs in response to treatment are a more useful immunologic surrogate of effectiveness in vaccine trials—an issue that needs further validation in future trials. Many questions remain regarding the value and optimal assessment of TILs, such as whether the TME needs to be assessed at specific locations (the tumor center or invasive margin), the relevance of specific subsets of TILs within the TME (e.g., CD3+ *vs*. CD8+ TILS), and whether changes in TILs overall or markers such as PD-1 correlate with response to ICI. Evaluation of changes in TILs in response to vaccination has the potential to be a useful tool in phase 1 trials to determine if vaccines generating a favorable anti-tumor response and to help guide dosing and schedule in early phase experience.

Though heterogeneous, the evidence reviewed here provides several targets for future investigations aimed at better understanding the role TILs may play as cancer vaccine endpoints. The most impactful data to date involves the use of Sipuleucel-T, a vaccine with proven biologic activity in prostate cancer, summarized previously. Significant increases were observed in multiple subtypes of infiltrating immune cells at the tumor interface while demonstrating active T-cell phenotypes, yet these changes did not correlate with a change in the metrics chosen to assess the peripheral immune response (52). Survival benefits have been demonstrated with changes of the TME and increased TILs, even while not simultaneously showing a detectable change in the assessment of the peripheral immune response. This data underscores the need to re-evaluate the validity of utilizing peripheral immune responses as a clinical trial endpoint in oncology patients treated with vaccination and suggests instead that evaluation of changes in TILs may be a better endpoint for treatment effectiveness.

When evaluating TILs, CD8+ lymphocytes are the most frequently evaluated subset. Diffuse CD8+ T-cell tumor infiltration seems to predict positive outcomes (46–48, 76), including improved survival in PDAC (59, 77) and CRC (69, 72), as well as halting disease progression with concomitant tumor regression on standardized radiologic evaluation in melanoma (46). Evaluating TILs subsets within the TME in addition to the change in the overall infiltrate following vaccination may provide an additional parameter to measure treatment response and prognosis.

Multiple studies observed the upregulation of PD-L1 after vaccination (59, 76, 77), which may translate into improved effectiveness of anti-PD-L1 therapy. This potential synergistic effect of vaccination and ICI therapy is of particular interest in immunologically “cold” or inactive tumors, where immunotherapy and vaccination have had limited success. PD-L1 staining after vaccination may also serve as a reliable way to predict the success of such a novel combination.

Future research involving both cancer immunizations and ICIs should aim to include data assessing TILs at multiple locations within the TME (with pre- and post-treatment matched biopsies) as well as information analyzing their subsets, subset ratios, and functionality with RNA-seq technology. Additionally, efforts should be made to standardize methodology for evaluating TILs to improve comparisons between trials. This data will help assess the ability and specific mechanisms by which cancer vaccines potentiate ICI targets and drive immunologically active TILs into the TME turning immunosuppressive “cold” into immunosupportive “hot” TMEs. Additionally, there have been newer studies which also describe the interactive role of natural killer (NK) cells in the TME (79). These developments have suggested a synergistic role of the cytotoxic T cells and NK cells that potentially have activity against various tumor cell types. Further research into the interaction among TILs and NK cells in larger tumor vaccination trials could potentially lead to better understanding of the cells contributing to the increased immunogenicity.

There are challenges to evaluating the tumor microenvironment in response to therapy in clinical trials. In general, getting matched biopsies for comparison is challenging in clinical trials even when heavily emphasized. Additionally, optimal timing of obtaining tissue is not always known when planning a trial and needed tissue collection, handling, and processing procedures create logistical challenges resulting in data heterogeneity. Tumor heterogeneity also creates a challenge in directly comparing pre- to post-treatment samples, potentially necessitating larger samples sizes to demonstrate a clinically meaningful difference in TIL infiltration. Emerging technologies, such as T-cell specific PET imaging modalities, may offer a solution to reliably obtaining a more complete evaluation of the local immunologic response to vaccination while overcoming operational tissue sampling challenges.

## Concluding remarks

The studies included in this review suggest a potential role for the quantification of TILs within the TME in future vaccination and active immunotherapy trials, when feasible, as a biomarker for clinical activity and to help guide the use of vaccines in novel combination immunotherapy trials. In this effort, special attention should be directed at the subtypes, and ratios of subtypes of TILs within the TME and surrounding stroma pre-, during, and post-treatment. Development of consensus methods for evaluating TILs and reporting results, while integrating novel T-cell specific PET imaging modalities will improve the validity and reproducibility of results and allow for improved comparison of results between trials.

## Data availability statement

The original contributions presented in the study are included in the article/supplementary material. Further inquiries can be directed to the corresponding authors.

## Author contributions

All authors contributed equally to this paper. They all performed a significant contribution that would warrant authorship. All authors contributed to the article and approved the submitted version.
